# A Solution for Sustainable Utilization of Aquaculture Waste: A Comprehensive Review of Biofloc Technology and Aquamimicry

**DOI:** 10.3389/fnut.2021.791738

**Published:** 2022-01-12

**Authors:** Ubair Nisar, Daomin Peng, Yongtong Mu, Yu Sun

**Affiliations:** ^1^Key Laboratory of Mariculture (Ministry of Education), Fisheries College, Ocean University of China, Qingdao, China; ^2^School of Management, Qingdao Agricultural University, Qingdao, China

**Keywords:** biofloc technology, waste utilization, nutritional composition, microbial interactions, sustainability

## Abstract

Biofloc technology (BFT) is gaining traction as a strategic aquaculture tool for boosting feed conversions, biosecurity, and wastewater recycling. The significant aspect of BFT is aquaculture with highest stocking density and minimal water exchange. It not only improves the water quality of a system by removing inorganic nitrogen from wastewater but also serves as a suitable feed supplement and probiotic source for cultured species. This technology is commonly used for shrimp and tilapia culture and can be used for both semi-intensive and intensive culture systems. Biofloc, when combined with formulated diets, forms a balanced food chain that improves growth performance. Nutrients in this system are continuously recycled and reused and form an efficient alternative system in aquaculture. In addition to the reduction in water exchange, it is also considered as a bio-security measure, since it prevents entry of disease from outside sources. Aquamimicry is an innovative concept that simulates natural estuarine conditions by developing copepods that act as supplementary nutrition especially for shrimp culture. The review highlights the process, significance, and development of BFT, its microbial interactions, nutritional value, transition from biofloc to copefloc, and concept of aquamimicry to sustainably improve aquaculture production.

## Introduction

With almost 7.8 billion people on earth, the demand for aquatic food is ever increasing; hence, the need for horizontal and vertical expansions of aquaculture production systems is highly recommended. At the same time, intensification of systems should be sustainable with more ecologically sound management practices. Moreover, the intensification of culture systems is likely to generate a tremendous amount of carbon-based pollutants causing toxic effects and environmental risks (1). The risks of these toxic discharges can further be reduced by continuously replacing pond water *via* exchange (2). Another approach for successfully removing major toxic pollutants without environmental concerns is recirculating aquaculture system (RAS) technique in which only 10% of total volume is replaced daily (3), but because of its high operational costs, the technique is hardly adopted. Therefore, there is a dire need for low cost and environmental-friendly technology for large-scale adoption. Therefore, an efficient alternative system that is environment friendly and with low operational costs was established and called “Biofloc Technology (BFT)” with which water nutrients are continuously recycled and reused. This sustainable approach grows microorganisms in a culture medium and promotes minimum water exchange. Microorganisms play an important role by uptaking nitrogenous compounds producing microbial proteins, which assist the system in maintaining water quality. The system is more economical and profitable, as it increases culture feasibility by decreasing FCR.

Aquaculture is a growing industry, and the vital component of the subject is feeding aquatic organisms under controlled conditions. Feed is a vital component in aquaculture and can hamper the expansion of the industry by dependence on fish meals and fish oil (4, 5). Such ingredients are one of the prime constituents of feed for commercial aquaculture (6). Feed costs in aquaculture represent at least 50% of total variable cost that is mainly due to high priced protein components in commercial diets (7). BFT in aquaculture uses food waste and organic matters produced during the production cycle through propagation of microorganism, which are developed using an external carbon source and high aerations (8). This technology has successfully demonstrated profitable results in fish and crustacean farming along with the production of value-added microbial proteinaceous feed for aquatic organisms. The specific objective of the study is to comprehensively review a study that has been conducted on the concept of biofloc technology and aquamimicry, which can further be disseminated to a global audience for better understanding of the concept and its practical applications in aquaculture. The study also signifies status and development in both concepts for sustainably improving aquaculture production using limited resources and cultivating commercially important species with fewer aquaculture generated wastes.

## Biofloc Technology

The biofloc system was initially developed for pollution-free and cost-effective productions to improve the environment, usually in areas where water is scarce. It has been an alternative for sustainable aquatic production in which bacteria converts fish waste to biomass (biofloc), eventually improving water quality through the addition of extra carbon to the aquaculture system, leading to minimal water use. Usually, in brackish water ponds, almost 70–80% of fed proteins go to waste in the form of nitrogenous metabolites. Thus, manipulating C:N ratio in aquaculture ponds encourages the uptake of this inorganic nitrogen into a microbial protein known as biofloc. If in the system, perfect balance of carbon and nitrogen in the solution exists, ammonium and other nitrogenous wastes will be converted into bacterial biomass (9). Furthermore, by adding a carbohydrate source to a culture pond, microbial proteins assist heterotrophic bacterial growth and nitrogen uptake (10, 11). It is essential to maintain a carbon-to-nitrogen ratio above 10 in a system by adding carbon source organic materials like molasses, starch, and wheat flour, or by reducing protein levels of the feed that increases the activity of heterotrophic bacteria produced (12–14). This eventually leads to the production of microbial proteins, and improves water quality and serves as a source of dietary proteins to cultured shrimps or fish (12).

The BFT was initially started in early 1970s at French Research Institute for Exploration of the Sea, Oceanic Center of Pacific where the concept was used for culturing various penaeid species including *Litopenaeus vannamei, Litopenaeus stylirostris, Penaeus monodon*, and *Fenneropenaeus merguiensis* (10). The BFT yields an economically viable system supporting high stocking density and biosecurity. The technology, which is practiced in a closed system, has the advantage of minimal environmental risk as used water is not released in natural water bodies like estuaries, lakes, and rivers, preventing eutrophication and loss of natural resources. C:N ratio manipulation develops dense bacterial communities, making a system dominated by bacteria rather than algae, promising a healthy rearing system and an approach for disease prevention. The BFT has been successfully implemented in aquaculture, especially in shrimp farming, because of its economic, environmental, and marketing advantages over conventional culture systems, making it a low-cost sustainable technology for sustainable future aquaculture development (5, 15). The technology provides higher degree of biosecurity and higher environmental control. It enhances the immune system (16) and contributes positively for strengthening the status of cultured species. It also has a favorable effect on the immunological response of *Litopenaeus vannamei* raised in a biofloc system, resulting in greater resistance to infectious myonecrosis virus (IMNV) (17) and Vibrio (18).

### Process of Biofloc Formation

**Figure 1:**
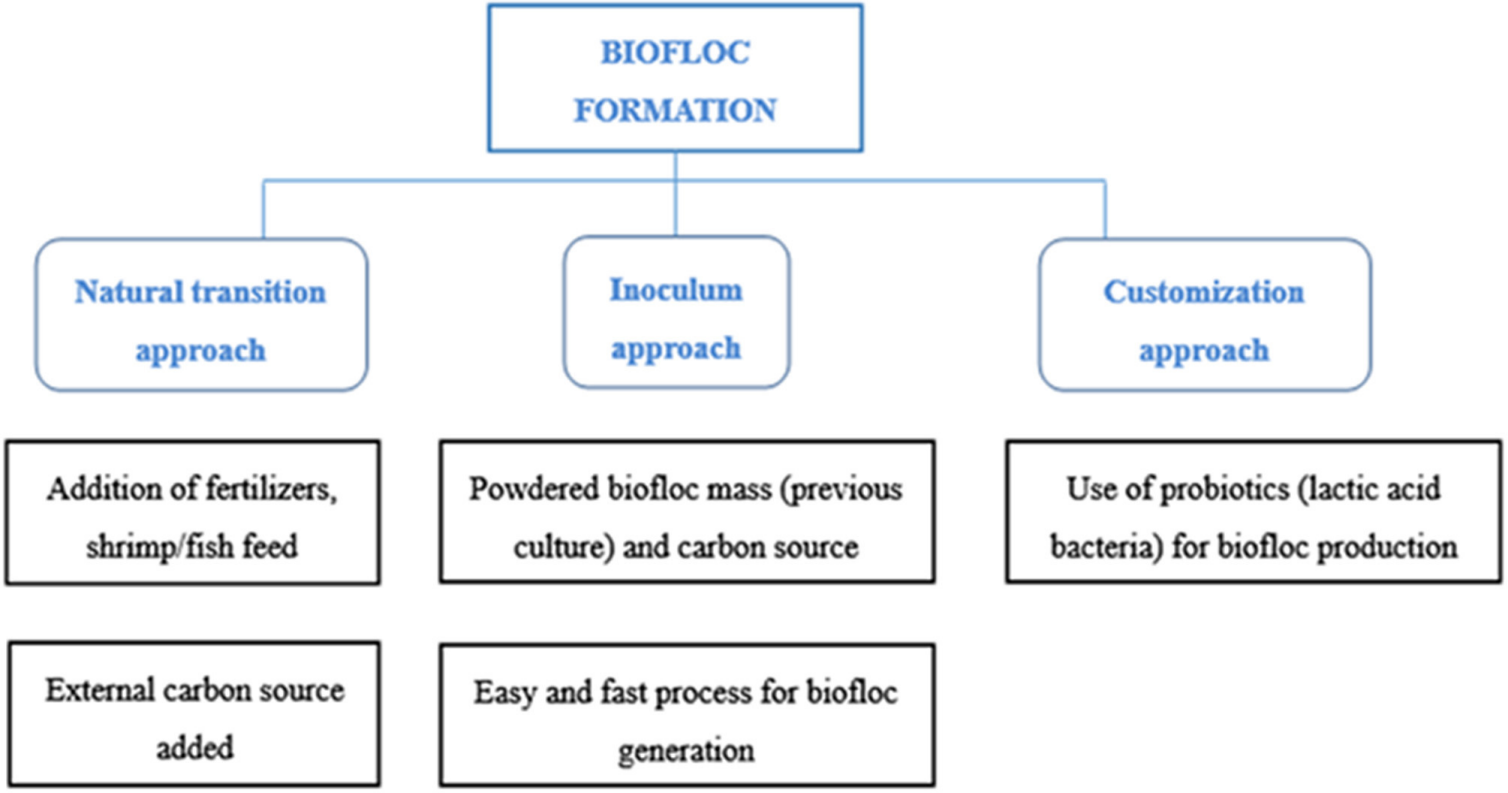
Schematic diagram of various methods to generate biofloc based culture practices.

High-density polyethylene (HDPE)-lined ponds with well-prepared dikes are most preferred for large-scale biofloc fish or shrimp production. HDPE-lined ponds with sufficient elevation and central drainage are used for effective biofloc-based farming. The BFT can be conveniently applied in re-circulatory aquaculture or raceway systems either by *in situ* inclusion or *ex situ* floc production through an activated sludge system and by putting the harvested biofloc in the production system. The system is adequately agitated and aerated to keep the microbial floc in suspension. For the development of biofloc, a small nitrogenous source (fish feed or urea) is added to previously filled water tanks. Then, a healthy carbon source like starch, molasses, or wheat flour is uniformly spread on the surface of water. To create a microbial mass, clay is softened and passed through a sieve (53-μm sized particles or less) and then added to the microbial reservoir. In addition, the use of farm wastewater containing nitrogenous wastes is helpful as an inoculum. The addition of 20 g of clay, 10 mg of ammonium sulfate, and 200 mg of carbonaceous organic matter such as molasses stimulates biofloc formation in 1 L of water (19). The primary inoculum in biofloc production cycle improves microbial mass formation. However, care should be taken before using any commercial inoculum for this purpose. Heavy aeration is required for keeping the floc under suspension. The use of clay and water rich in biofloc production cycle as the primary inoculum improves microbial mass formation in the new culture system (20). Heterotrophic bacteria are more active than other bacteria because of the presence of the carbonated organic matter, which assists the removal of carbon and nitrogen from the water, and produces microbial biomass (13). This microbial biomass is attached and fed by other organisms in the water forming biofloc (14). Physical and chemical parameters like temperature, oxygen, pH, alkalinity, total nitrogen, ammonium, nitrite, and nitrate should be measured; subsequently, appropriate responses should be adopted quickly (21, 22). As explained in Figure 1, there are three major approaches for successfully generating biofloc.

### Approaches of Biofloc Generation

#### Natural Transition Approach

In this approach, autotrophs are generated through addition of fertilizers, fish/shrimp feed, and other ingredients. The autotrophs are further converted into heterotrophs by adding a carbon source and maintain the ratio of C:N (12–15:1). The amount of salinity and type of carbon used in the system affect the rate and duration of biofloc formation (13). It is observed that increasing salinity increases the density of biofloc formation, and the quality of the floc is determined by the type of carbon source (23). Furthermore, improved water quality and faster growth of heterotrophic bacteria are achieved by adding carbonaceous organic matter-like molasses to the aquaculture system without exchanging the water compared to the addition of complex carbohydrates like wheat flour. Color transition from green to brown can be observed, with the floc building up during the process. Disadvantages of this approach are it is a time-consuming process, and simple conversion of autotrophs to heterotrophs takes several days.

#### Inoculum Approach

The main idea of this technique is to inoculate new biofloc based culture water with it after evaluating the compatibility of the previous crop in terms of nutrients availability and water quality. Fermented products of the carbon source (rice bran, molasses, etc.) are to be aerated with the source water for at least 24–48 h for biofloc generation (20). Finally, the cultured biofloc mass produced earlier is dried and developed into a powder form. This powder is then stored and dissolved with the carbon source to be added in the form of fermented products. The benefit of this approach over natural transition is that it is a time-saving process; therefore, biofloc can be generated in a very short duration.

#### Customization Approach

This method of biofloc production is the most sophisticated one, as it involves blending of probiotics in the system for a healthier environment. Previously, it has been discovered that excessive amount of chemical compounds and antibiotics (oxytetracycline, sulphamethoxazole, chlortetracycline, amoxicillin, co-trimoxazole, and sulphadiazine) was used to suppress a disease outbreak in aquaculture (24–28). However, these compounds have not only been proved lethal for human consumption, causing health risks, but have also been proved to induce immune suppression. Different microorganisms (probiotics or microalgae) are capable of inducing better aquaculture production by modulating the immune system in many ways. Thus, the need for natural friendly alternatives and growth promoters has become important for healthier aquaculture. Then comes the role of probiotics, which are important to maintain a healthy environment and positively increase aquaculture production without causing any negative effect on the health of consumers (29). These probiotic strains are widely used in shrimp culture and can be blended in right combinations in biofloc to get the best floc results. The floc can be manipulated and prepared to contain naturally occurring bacteria capable of producing high concentration of enzymes. Bacterial probionts like lactic acid bacteria*, Pseudomonads*, and *Bacillus* are found to give better results in salmon and shrimp production by degrading the organic matter, reducing hydrogen sulfide and ammonia accumulation in the system (18). Bioremediation, a process that uses microbes for waste water cleaning, involves organic matter mineralization to carbon dioxide and maximizes primary productivity that stimulates shrimp production (30). To maintain a stable BFT, excess nitrogen is to be eliminated from the system by nitrification and denitrification processes, and exclusion of pathogens in the system by desirable bacterial species (11).

For successfully developing the culture cycle, water quality parameters like temperature, dissolved oxygen (DO), total suspended solid (TSS), salinity, and alkalinity are to be measured and monitored constantly. The recommended water quality parameters for tropical species (e.g., *Litopenaeus vannamei* and *Oreochromis niloticus*) of biofloc technology are shown in Table 1. In addition to maintaining water quality, rigorous turbulent mixing is also an essential requirement for a system to function well, and solids must be suspended in a water column at all times. Without proper mixing, biofloc forms piles and can rapidly consume dissolved oxygen in the system and can further lead to anaerobic zones releasing ammonia, hydrogen sulfide and methane. Solids can be removed from the system by regular flushing or by manually pumping sludge out from the pond center. Aeration has dual functionality in the system, it is used to supply oxygen and at the same time provides proper mixing (32). The most common aerator used in the pond culture is paddle wheel aerator supplying efficient oxygen, but it is not ideal for pond mixing (32). Various configurations of aeration equipment can be used for efficient mixing and aeration depending on the form of the biofloc system. In shrimp indoor raceways, a number of airlift pumps are used and placed at regular intervals for proper water circulation. Devices that circulate water at low head, such as low-speed paddle wheels and airlift pumps, can also be used. The BFT is not viable for areas with expensive electricity and unreliable power supplies (21).

**Table 1:** Ideal water quality parameters for successful biofloc technology (31).

**Parameters**	**Ideal range**	**Observations**
Dissolved oxygen (DO)	Above of 4.0 mg L^−1^ (ideal) and at least 60% of saturation	For correct fish, shrimp, microbiota respiration, and growth
Temperature	28–30° (ideal for tropical species)	Besides fish/shrimp, low temperatures (~20°C) could affect microbial development
pH	6.8–8.0	Values <7.0 is normal in BFT but could affect the nitrification process
Salinity	Depends on the cultured species	It is possible to generate BFT, e.g., from 0 to 50 ppt
TAN	<1 mg L^−1^(ideal)	Toxicity values are pH dependent
Nitrite	<1 mg L^−1^(ideal)	Critical parameter (difficult to control). Special attention should be done, e.g., on protein level of feed, salinity, and alkalinity
Nitrate	0.5–20 mg L^−1^	In these ranges, generally not toxic to the cultured animals
Orthophosphate	0.5–20 mg L^−1^	In these ranges, generally not toxic to the cultured animals
Alkalinity	More than 100 mg L^−1^	Higher values of alkalinity will help the nitrogen assimilation by heterotrophic bacteria and nitrification process by chemoautotrophic bacteria
Settling solids (SS)	Ideal: 5–15 mL L^−1^(shrimp), 5–20 (tilapia fingerlings) and 20–50 mL L^−1^(juveniles and adult tilapia)	High levels of SS will contribute to the DO consumption by heterotrophic community and gill occlusion
Total suspended solids (TSS)	<500 mg L^−1^	Ideal to SS

### Microbial Community in Biofloc

Biofloc is defined as a set of organic matters formed usually at high density as suspended particles (33–35), and it comprises 60–70% of organic materials, and combination of fungi, algae, bacteria, protozoa, rotifer, nematodes, and other inorganic substances (36). In a zero-water exchange system, water quality is maintained by two functional bacterial population, viz., heterotrophic ammonia- assimilative and chemoautotrophic-nitrifying bacteria (37, 38). As the culture progresses, the color changes from green to brown, indicating the transition from algal-dominated to bacterial biofloc-dominated system. The number of bacteria in biofloc ponds can be between 10^6^ and 10^9^ ml^−1^ of floc plugs, which contain between 10 and 30 mg dry matter, making the pond a biotechnological industry (39). Khanjani et al. (14) specified that the maturation of the biofloc can be determined by the number of heterotrophic bacteria per ml, which is 3.36 × 10^7^. The type of carbon source, salinity level, and species to be cultured are major factors determining the different group of organisms present in biofloc (40). The biofloc collected from *Litopenaeus vannamei* tanks contained 24.6% phytoplankton (dominated by diatoms like *Thalassiosira, Chaetoceros*, and *Navicula*), 3% bacterial biomass (two-thirds was Gram-negative and one-third was Gram-positive), a small amount of protozoan community (98% flagellates, 1.5% rotifers, and 0.5% amoeba), and 33.2% detritus; the remaining quantity of 39.25% was ash (16). However, the composition may vary within different culture systems. Of the total organic sludge, it is believed that only 2–20% is living as microbial cells, while the rest is total organic matter (41).

Some of the dominant bacterial species found in the biofloc system are *Proteobacterium, Bacillus*, and *Actinobacterium*, and traces of other bacterial species (*Roseobacter and cytophaga*) are also present in the system (42). For a bacterial species to run a cellular machinery, it needs nitrogen like ammonium ion and a carbon source such as sugar, starch, and cellulose to make it responsive to toxic ammonia from the aquaculture system. The ability to adhere to suspended particles and surfaces as well as the use of organic matter are important physiological properties of bacteria in biofloc. The role of bacteria here is to transform the toxic form of nitrogen to one that is toxic only at high concentrations by the process called nitrification. This bacterial growth *via* promoted nitrogen uptake decreases ammonium concentration more rapidly than nitrification (38). The rapid occurrence of heterotrophic bacteria is due to the growth rate and microbial biomass yields per unit substrate of heterotrophs being a factor 10 higher than that of nitrifying bacteria (38). The microbial biomass yield per unit substrate of heterotrophic bacteria is about 0.5 g biomass C/g substrate C used (43). The optimum C:N ratio in an aquaculture system can be maintained (C:N ratio 15–20:1) by adding different locally available cheap carbon sources and/or reducing protein percentage in feed. Under optimum C:N ratio, inorganic nitrogen is immobilized into bacterial cell, while organic substrates are metabolized. Bacteria have a nutrient conversion ratio as high as 50% and possess fast multiplication rates converting toxic product in the biofloc system into highly nutritious and useful microbial protein (42). Therefore, the biofloc system could be considered as a microbial ecological sequence model. It is reported that heterotrophic microbial communities like *Pseudomonas, Bacillus, Sphingomonas, Micrococcus, Nitrospira, Nitrobacter*, and yeast maintain water quality and manages the physiological health of cultured species in a biofloc system (44).

Carbon-nitrogen ratio in biofloc systems plays a vital role in the restriction of toxic inorganic nitrogenous compounds into beneficial bacterial cells acting as a source of food for cultured organisms (10). Immobilization of inorganic nitrogen takes place when the C:N ratio of the organic matter is higher than 10 (45). In a biofloc system (intensive culture), the growth of heterotrophic bacteria is restricted by dissolved organic carbon. The population of the heterotrophic bacteria in the system is stimulated by adding a supplementary carbohydrate source or reducing the feed protein level manipulating the C:N ratio and creating a demand for nitrogen (ammonia). The generated ratio of organic carbon and inorganic nitrogen reflects the requirement and composition of heterotrophic bacterial cells. Substantial development of useful microbial growth and fixation of ammonia in a culture medium are possible by directly or indirectly adding a carbon source in limited discharge systems (changing C:N ratio) (11, 15, 37, 46). Thus, manipulating the C:N ratio results in shift from an autotrophic to a heterotrophic system (4, 10).

### Nutritional Value of Biofloc

Biofloc systems possess a dynamic nutritional value and can be used as a complete aquatic food source and supply bioactive compounds (47). There are many factors that can affect nutritional value such as food preference, floc density in water, and ability of animals to ingest and digest microbial proteins (38). The heterotrophic bacterial population in a biofloc system produces single-cell proteins, which act as a source of food for carps, shrimps, and tilapia (48–50). Qualitatively, biofloc contains crude protein (50%), crude lipid (2.5%), fiber (4%), ash (7%), and 22 kJ g^−1^ energy (51). It is observed that the nutritional value of biofloc depends on the biochemical compounds present, particle size, and digestibility of cultured organisms. Ekasari (17) found that particle sizes of <100 μm and more than 45 μm are more promising for *L. vannamei, Oreochromis niloticus*, and *Perna viridis* because of higher nutritional values. The highest level of proteins and lipids are found in the particle size of >100 μm, whereas the amino acid concentration is found highest in <45 μm. Table 2 presents various studies conducted on different aquatic species by adding different carbon sources and microbes reported.

**Table 2:** Studies with different carbon sources, species, and microbes reported by different authors.

**Carbon sources**	**Species**	**Microbes reported**	**References**
Molasses	*L. vannamei and P. monodon*	–	(49)
Starch	*L. vannamei and M. rosenbergi*	–	(52)
Wheat flour Tilapia	*O. niloticus*	*A.Protozoa, B. Rotifera C. Oligochaeta A. Paramecium, Tetrahymena and Petalomonas*	(51)
Cellulose	*Tilapia*	–	(15)
Dextrose	*Litopenaeus vannamei*	–	(53)
Tapioca starch	*M. rosenbergii*	Rotifers: Lecane, Trichocerca, Polyarthra and Asplanchna. Oligochaeta: Tubifex	(54)
Acetate	*Macrobrachium rosenbergii*	–	(11)
Wheat bran and molasses	*Farfantepenaeus paulensis*	Phytoplankton, periphyton, zooplankton, microbial floc and benthic macro invertebrates	(55)
Wheat bran + Molasses	*F. paulensis*	*Lactobacillus* spp.	(56)
Wheat bran + Molasses	*F. brasiliensis*	–	(35, 57)
Sugarcane molasses Tapioca flour wheat flour	*Litopenaeus vannamei*	*Vibrionaceae, Enterobacteriaceae Alteromonadaceae* and *Micrococcaceae*	(58)
Molasses + dextrose + rice flour	*Litopenaeus vannamei*	–	(59)
Molasses, Molasses+ rice powder	*Oreochromis niloticus*	Tintinids, Ciliates, Copepods, Spirulina and Nematodes.	(60)
Wheat bran	*Litopenaeus vannamei*	–	(61)
Molasses + wheat flour + Starch	*Litopenaeus vannamei*	–	(13)
Sugar beet molasses:	*Cyrinus carpio*.	*Lactobacillus* spp.	(62)
Molasses + palm sap	*Litopenaeus vannamei*	–	(63, 64)
Wheat flour and Molasses Tilapia	*O. niloticus*	–	(65)

It is reported that the feed conversion ratio (FCR) values of a fish culture in the biofloc technology can be reduced to 1.2–1.29 from 1.52 in clear water (22). In clear water treatments, feed efficiency was recorded as low as 66.81%, whereas the same was 84.26% in BFT (22). Ballester et al. (55), in his study on the BFT, used wheat bran and molasses as carbohydrate source and revealed the nutritional breakdown of biofloc as 30.4% crude protein, 4.7% crude fat, 8.3% fiber, 39.2% ash, and 29.1% nitrogen-free extract. Khanjani et al. (13) used molasses, starch, wheat flour, and their mixtures as carbon sources and suggested that experimenting with different carbon sources changes the nutritional composition of the biofloc produced. Ray et al. (40) revealed that in super-intensive shrimp culture systems, an environment friendly plant-based diet can produce results similar to those from fish-based feed in the BFT, and that controlling the concentration of particles could improve water quality and shrimp production. Samocha et al. (66), in his study on 120 days of *L. vannamei* biofloc culture, reported 92, 81, and 75% survival with stocking of 150, 300, and 450 shrimps/m^2^, respectively, and detected no significant changes in FCR when feeding *L. vannamei* with different percentages of CP diets; he claimed that floc biomass provides a complete source of cellular nutrition to the system. The exact mechanism of growth enhancement by microbial flocs is unknown, but it is believed that continuous consumption of native proteins without previous treatments (56) could possess a growth factor enhancing the growth in the system. Biofloc improves ingestion and digestion of supplied feed and provides cellular nutrition to cultured organisms (67). Emerenciano et al. (57) stated that the presence of biofloc in the brood-stock diet of *Litopenaeus vannamei* and *Penaeus stylirostris* improved reproductive performance and egg quality. In his study, *L. vannamei* females were cultured under biofloc conditions and fed with fresh food, and later showed higher egg production and better spawning rates, and contained higher levels of highly unsaturated fatty acids (HUFAs) in eggs.

### Wastewater Reduction by BFT

In addition to improving target species production, the BFT can help conserve the amount of water required in aquaculture. Biofloc, compared to traditional aquaculture practices, provides a more sustainable approach with minimum water exchange and lower feed intake, transforming it into a low-cost sustainable technology for aquaculture development (5, 15). Traditional aquaculture systems with regular water exchange require up to 80 m^3^ kg^−1^ shrimp, but intensive shrimp farming systems with zero exchange require just 1–2.26 m^3^ kg^−1^ shrimp (38). When compared to a recirculating aquaculture system (RAS), biofloc systems based on tilapia production utilized about 40% less water (68). Majority of studies using the BFT found that nitrogen and phosphorus waste in this system could be decreased further, supporting the importance of the system in improving aquaculture productivity and minimizing environmental effects from aquaculture production systems (68, 69). Heterotrophic bacteria in a biofloc system assimilate inorganic nitrogen compounds at a faster rate than denitrifying bacteria, resulting in a 10-fold increase in microbial biomass (38). As a result, if there are enough organic carbon sources available, heterotrophic bacteria can immobilize ammonia in biofloc in a matter of hours or days (38). Although heterotrophic bacteria are primary nitrogen conversion agents, the biofloc system also aids in nitrification, phototrophic nitrogen uptake, and denitrification (17). Farm biosecurity and biofloc technology are two key aspects that must be considered for long-term intensive aquaculture sustainability. The BFT improves biosecurity by limiting water exchange, increasing environmental control, creating biological and physical (indoor) barriers to infections, and boosting the immune system.

### Effect of Biofloc System on Aquaculture Microbiome

Wasielesky et al. (70) studied various aspects like endurance, growth, feeding pattern, and feed conversion ratio (FCR) of *L. vannamei* juveniles in a biofloc system, and positive association was observed between growth of shrimp and protein content, depicting the benefits of the BFT for the culture of white-legged shrimp (*L. vannamei*). Another study on white-legged shrimp in the BFT (8) showed higher growth rates, improved water quality, and increment in the final body weight of the shrimp. The comparative analysis of *Farfantepenaeus brasiliensis* post larvae when stocked with and without a biofloc system showed better growth and survival in the biofloc system because of high nutritional environment (34). Pinto et al. (71), in his study on culturing the *L. vannamei* utilizing artificial seawater in the BFT, concluded that the BFT system was zootechnically and financially viable. Ferreira et al. (30), in his study on the BFT, reported the presence of *Bacillus* species demonstrating the probiotics nature of the system. Huang et al. (72), in his study on *L. vannamei* in the BFT, observed an increase in the concentration of *Actinobacteria, Alteromonadaceae*, and *Rhodobacteraceae* and a decrease in the number of *Cyanobacteria, Mycoplasmataceae*, and *Vibrio*, indicating t the higher C:N ratio in the system responsible for enhancing and promoting beneficial bacteria and ultimately suppressing harmful pathogens. Hence, from the above studies, it can be explained that the BFT could be used as an innovative strategy to develop and grow enriched probiotic microbes for improving health and enhancing growth of cultured species (73).

It is of prime importance to conduct research on species for the BFT, which are commercially important and have a social interest. The only sole member of the freshwater prawn species having published literature associated with the BFT is *Macrobrachium rosenbergii*, allowing it to be used commercially. Considering the research on shrimps includes species like *L. vannamei* (74), *Penaeus monodon* (46), *Litopenaeus setiferus* (75), *Fenneropenaeus merguiensis* (76), and *Farfantepenaeus paulensis* (77). There are many other species of the family to which the BFT has successfully extended such as *F. brasiliensis* (34), *F. duorarum* (57), *L. stylirostris* (34), *L. schmitti* (78), *Marsupenaeus japonicus* (42), and *Fenneropenaeus indicus* (79).

*Oreochromis aureus* (10), *Oreochromis mossambicus* (39), and *Oreochromis niloticus* (51) are among the freshwater fish species studied using the BFT. The technology has also been extended to two major commercially important carps, i.e., *Catla cattle* (80) and *Labeo rohita* (50). For the ornamental fish industry, various species have also been studied using this technology like *Poecilia reticulata* (81), *Scatophagus argus* (82), *Carassius auratus* (83), and *Pseudotropheus saulosi* (84). Some other commercially important species that have been studied using the BFT are shown in Table 3.

**Table 3:** Applications of the biofloc technology (BFT) in species based on their bibliographic references.

**Species**	**Family**	**Bibliographic references**
*Litopenaeus vannamei*	Penaeidae	McIntosh (74), Tacon et al. (67), Burford et al. (49), Hari et al. (46), Wasielesky et al. (70), Ju et al. (16)
*Penaeus monodon*	Penaeidae	Hari et al. (46)
*Penaeus semisulcatus*	Penaeidae	Megahed (85)
*Penaeus merguiensis*	Penaeidae	Aquacop (76)
*Marsupenaeus japonicus*	Penaeidae	Zhao et al. (42)
*Litopenaeus stylirostris*	Penaeidae	Emerenciano et al. (34)
*Farfantepenaeus brasiliensis*	Penaeidae	Emerenciano et al. (34)
*Litopenaeus setiferus*	Penaeidae	Emerenciano et al. (75)
*Catla Catla*	Cyprinidae	Prajith (80)
*Labeo rohita*	Cyprinidae	Prajith (80), Mahanand et al. (50)
*Tinca tinca*	Cyprinidae	Carbo and Celades (86)
*Carassius auratus*	Cyprinidae	Faizullah et al. (83),
*Cyprinus carpio*	Cyprinidae	Sarker (87), Najdegerami et al. (88)
*Labeo victorianus*	Cyprinidae	Magondu et al. (89)
*Macrobrachium rosenbergii*	Palaemonidae	Crab et al. (11), Prajith (80)
*Oreochromis aureus, O. niloticus*,	Cichlidae	Avnimelech (10)
*Etroplus suratensis*	Cichlidae	Thilakan et al. (90)
*Clarias gariepinus*	Cichlidae	Dauda et al. (91), Putra et al. (92)
*Poecilia reticulata*	Cichlidae	Sreedevi and Ramasubramanian (81)

### Economic Aspects of BFT

To make any aquaculture venture profitable, there is a need to reduce production costs and increase profitability. The key deciding factors for the aquaculture industry to sustain in the long-run are feed cost and environmental protection (21). In this technology, the carbon source used is mainly a by-product that is derived from either the plant or animal food industry and is locally available. Before the stocking of post larvae and during the grow out culture, cheap sources of carbohydrates like molasses and plant meals are applied in the system to provide food for initial stages of growth and maintain the C:N ratio.

The biofloc system is responsible for increasing the growth rate and reducing the feed conversion ratio, which eventually increases profitability and reduces aquaculture costs (22). Megahed (85) found that the per-kilogram production cost of green tiger shrimp (*Penaeus semisulcatus*) and tilapia was reduced by 33 and 10% (93), respectively, using the BFT depending on diet, species, and price of carbohydrates. The biofloc system is more successful in increasing growth rate and reducing culture period than the clear water system (94). Liu et al. (18) showed that shrimp yield can be increased, and that feed conversion ratio can be lowered by adding maize to stimulate biofloc growth in an integrated culture of shrimp, spotted scat, and water spinach. This process can further reduce the total phosphorous and total nitrogen in the cultured water. Similarly, Ekasari (17) demonstrated that biofloc-based polyculture of shrimps, tilapia, mussel and, seaweed resulted in higher growth rate and reduced waste nutrient and microbial biomass. The biofloc system reduces the cost of organic and inorganic fertilizers and covers the cost of carbon source. The BFT is also responsible for reducing water treatment expenses by 30%, and protein utilization efficiency is double when compared with conventional water treatment technologies (5, 21).

When investing in a new or experimental technology, it is necessary to adapt it to the local environment until the production process can be standardized and survival can be maximized (95). Rego et al. (96), in his study, estimated the financial viability of *Litopenaeus vannamei* culture in the BFT and found that per hectare BFT operating costs are 10 times higher than those of the conventional aquaculture practice. Among the total variable costs, feed contributed highest in both systems, accounting for 54% in the BFT and 79% in conventional (96). Feed was found to be the most important variable cost for intensive shrimp farming in Asia (97), accounting for between 23 and 46 % of expenditures. Rego et al. (96) found that in the BFT, total production cost per cycle and total production cost per year were US$ 33,294.87 and 124,369, respectively, with stocking density of 113 shrimp per m^−2^. In terms of production cost, the BFT system generated an annual operating profit of US$ 51,871.54 per productive hectare, which is 141% greater than that of the conventional approach. In comparison, Hanson (98) estimated an initial expenditure of around US$ 992,000, with total production cost equivalent to US$ 983,950.00 year^−1^, for a super-intensive BFT system operating with densities of 500 shrimps per m^−3^ in 10 tanks of 500 m^3^ each, located in Texas, United States. The high overall production cost of the BFT system compared to the conventional system is justified not only by the highest feed expenditure but also by the high labor work and energy values that significantly contribute to this variance. Hargreaves (32) stated that the greater energy consumption in the BFT system is due to the shrimp and heterotrophic bacteria in biofloc having high oxygen demand, necessitating the need for artificial aeration in a minimum ratio of 25 HP ha^−1^, as opposed to only 3 HP ha^−1^ in the conventional system.

### Biofloc Technology for Sustainable Aquaculture

The concept of BFT goes against the popular understanding that pond water must be clean; thus, convincing farmers to use the approach is a big challenge (21). It is also because of the lack of perfect technology, justifying BFT technology and persuading farmers to implement it is more challenging than with traditional approaches. Several considerations, on the other hand, encourage the use of the technique. To begin with, water has become scarce or expensive to the point that aquaculture development is being hampered. Second, most nations have laws prohibiting the discharge of contaminated effluents into the environment. Finally, significant outbreaks of contagious diseases prompted the implementation of overly restrictive biosecurity measures, such as lowering water exchange rates (21). Monitoring of ponds is a critical part of implementing the technology in aquaculture. The BFT is not yet entirely predictable, making its implementation at the farm level riskier.

Water quality monitoring including determining and stabilizing the total concentration of suspended solids, settling solids, the number of aerators, their type and location in ponds are important for the successful implementation of the technology (5). Various findings have revealed that BFT have the ability to control dangerous chemicals in aquaculture systems, which, if left intact, can reduce productivity (21, 99). The BFT system has been tested for multi-species production systems, like tilapia with vegetables, shrimp with microalgae and seaweed, producing favorable results (100). Aquaculture is a sustainable technique that focuses on environmental, social, and economic concerns as it grows, according to the BFT. The BFT is developed on the principle of recycling and reusing nutrients, particularly nitrogen, into microbial biomass, which may either be utilized by cultured organisms or collected and processed into important feed nutrients (101). The development of such an approach necessitates careful modification and execution, requiring additional research and information from researchers, producers, and customers in order to establish a base for this method, which ultimately is the foundation of sustainable aquaculture.

### Integrated Multi-Trophic Aquaculture Using Biofloc

While the BFT helps in the maintenance of adequate water quality suitable for the survival and general well-being of reared aquatic species without the use of massive water volumes and exchange rates, the accumulation of total suspended solids (TSSs) and organic matter in rearing units may cause a number of environment problems. In the BFT, an integrated multi-trophic aquaculture (IMTA) system can be set up in which the waste of a new organism is used as feed for another (102). The system allows filter feeding organisms like *Oreochromis* sp. and *Mugil liza* to incorporate in the culture system, so suspended particles and organic debris that accumulate at the bottom of rearing units are absorbed (102). However, the organisms should not compete for food and space with co-cultured species. The species to be cultured is a crucial consideration when building a biofloc system. Biofloc systems function best with species that can benefit nutritionally from direct floc ingestion. Biofloc systems are also best for species that can withstand high sediment concentrations in water and are tolerant of poor water quality. Physiological changes in shrimp and tilapia allow them to absorb biofloc and digest microbial protein, and to take advantage of biofloc as a food source (82). Earlier studies have revealed an increase in N and P recovery, growth performance, profitability, yield, and immunity in integrated culture of shrimps and Nile tilapia (Oreochromis niloticus) when stocked at various stocking densities (5, 103). Borges et al. (102), in his study on integrated polyculture of BFT in mullet (*Mugil liza*) and white shrimp (*Litopenaeus vannamei)*, revealed that the organic matter in rearing units were utilized by the mullet when raised in same units or two different units with 41 days of culture. When tropical fish species like tambaqui (Colossoma macropomum) were cultivated in the BFT, no improvement in growth or productivity was noted when compared to conventional culture techniques; however, major water problems like turbidity and amount of nitrite increased in the system (102).

There are many methods that can be adopted for increasing the efficiency of BFT when culturing a particular species or a combination of species. One of them is to use lined ponds like those used in countries like Australia, Indonesia, and Malaysia for commercial shrimp culture (15). The basic strategy is to employ small (0.5 to 1.5 ha) ponds lined with plastic (30- to 40-mil HDPE) and aerate vigorously (28–32 hp/ha) with paddle wheel aerators to keep floc in suspension (15). This system requires high stocking density of PL (125–150/m^2^) to maximize yield. The culture is carried out usually for 90–120 days with maximum daily feed of 400–600 kg/ha before harvesting. This technique of lining shrimp ponds has produced tremendous yields of 20–25 metric tons/ha per crop (15). Another approach is the use of greenhouse raceways for shrimp, which member universities of the old US Marine Shrimp Farming Consortium developed for intensive lined raceways in standard greenhouses (100 feet long × 25 feet wide), building on the intensification of lined, outdoor shrimp ponds. If supplemental heat is given, these greenhouses can be located inland to avoid expensive coastal land, and in temperate climates (93). The stocking density of these shrimps varies from 300 to 500 PL/m^2^, and yields an output of 3–7 kg/m^2^. Higher yields of around 10 kg/m^2^ are also found when supplied with pure oxygen supplementation (93).

### Applications of Biofloc Technology

It was in the mid-1990s when first commercial uses of BFT in shrimp aquaculture were reported in Belize. The area of culture ponds were around 1.6 ha producing 11–26 mt of shrimp in each cycle (35). Commercial large-scale and small-scale BFT-based shrimp farms are now spreading across Indonesia, Malaysia, Thailand, South Korea, China, and India (35, 80). It is reported that the commercial size of a biofloc pond varies between 0.1 and 2 ha with proper aerators (paddle- wheel) and aspirators in order to aerate the system well and allow particles to be in suspension for proper mixing (34). The technology often leads to higher productivity without affecting the environment. Production intensity in BFT ponds is far more higher than that in non-BFT ponds, like a tilapia culture in the BFT system that exhibited higher growth and quality. The BFT improves output and productivity by assisting in the supply of high-quality fish juveniles, which is one of the most significant inputs in the production process. The culture system contributed around 45% higher production and individual weight gain than those without BFT (51). In Indonesia alone, the technology is used by almost 20–25% of farmers with an average pond area of 0.5 ha and produces more than 30 metric tons of output per cycle. High-density polyethylene (HDPE) sheets are used for lining ponds with an aeration capacity of 28 hp ha^−1^ (56). While full HDPE-lined ponds in Malaysia generated an average of 17–23 metric tons of produce every cycle, ponds with lining in dikes had a much lower output of 12 metric tons per cycle (56).

Furthermore, biofloc systems may be created and implemented in conjunction with other forms of food production, resulting in more productive integrated systems that seek to produce more food and feed from the same amount of land with less input. By boosting the reproductive performance of aquaculture animals and increasing the immunity and robustness of larvae, the BFT might help sustain the supply of high-quality seeds (17, 35).

## The Concept of Aquamimicry

Biofloc is being considered as a sustainable technology and cost effective method for controlling waste generated in the aquaculture system, but it also has some disadvantages which deter shrimp farmers from using this. The major drawback of this technology is the need for continuous aeration to suspend the waste generated in the system so that active metabolism by bacteria to generate proteins could take place. Second, the drop in pH and alkalinity due to nitrification and the need to add sufficient carbon are all factors that need to be monitored closely compared with conventional methods of shrimp farming (104). These constraints lead to the development of a new novel technology called the copefloc technology that relies on the natural production of copepods in the system that are further eaten by stocked shrimps. The technique negates the use of external feed source or any rigorous churning and oxygenation in the culture system (105). Zooplankton copepods produced during the process are very advantageous, convert energy in the food chain, act as a source of food for marine animals, and perfect nutrient recyclers (106). Shrimps, when fed with these copepods, showed better growth and improvement in survival rate (107) because of better biochemical composition of the plankton (108).

The orders harpacticoid, calanoid, and cyclopoid are the three major candidate species of copepods for aquaculture production and usually dwell in fresh, marine and estuarine environments (109). Harpacticoid copepods are epi-benthic and have superior nutritional attributes compared to Artemia and rotifers (108). The advantage of harpacticoids over other rotifers is that they can grow at high densities (110). Cyclopoid copepods are rarely used in aquaculture, and a density of ~5,000/L is possible to achieve in cultures (111). It is due to its difficulties in harvesting nauplii and lack of storage possibilities for egg that the culture practice is not standardized in spite of high nutritional profile. Calanoid copepods are pelagic and the natural prey for fish larvae (112).

An innovative concept of *in-situ* waste assimilation creating blooms of zooplankton (copepods), enhancing the growth of beneficial bacteria and acting as a good source of supplementary nutritional form in shrimp culture, is aquamimicry (113). In aquamimicry, a carbon source such as fermented rice bran (FRB) is added with some probiotics that generate phytoplankton and zooplankton blooms and simulate natural pond conditions. These planktons acts as a supplemental nutrition and improve water quality in fish and shrimp cultures (114). As per Romano (109), FRB is made by adding water, some hydrolyzing enzymes, and probiotics to rice bran powder and is then allowed to soak overnight. The pH of incubation water should be in the range of 6–7 and adjusted if necessary. The prepared mixture is allowed to ferment for 24 h and then added to cultured ponds at the rate 500–1,000 kg ha^−1^ (115). If the rice bran used is in powdered form, it is added gradually to the pond, and if the crumble form is used, its supernatant/juice is added to the pond, and solid bran particles are fed to fishes in the bio filter pond. The dominance of copepods in the ponds can be observed within a week of application. Now, once the zooplankton is ready, the ponds are stocked with shrimp post larvae at the stocking density of 20 PL m^−2^ (115). In order to sustain the copepod bloom, the stocked ponds are regularly seeded with FRB at the rate of 10 kg ha^−1^ every month (115). The stocked shrimps are provided with the supplementary nutrition, as they feed easily on particles of FRB.

For high stocking intensive culture, the concept of aquamimicry can also be adopted; in that case, a central drainage system is developed from grow-out pond to sedimentation pond. In grow out ponds, fishes like catfish and milkfish are grown, which churn up the detritus promoting the growth of oligochaete worms rich in essential amino acids, such as lysine and methionine and can be eaten by the fishes (114). Furthermore, in bio filter ponds, fishes like tilapia can be grown, which can further reduce the waste in the water coming from sedimentation ponds and can be reused and pumped back in the grow out. To maintain water quality in grow out ponds, additional probiotics should be added every month. In order to accumulate sediments, a sedimentation pond should be constructed deeper than grow out and stocked with catfish/milkfish (bottom dwelling) at lower densities for pond cleaning. The sediments accumulated from grow-out ponds generate worms and other benthic invertebrates that are further eaten by fishes. Now, from sedimentation ponds, the water overflows to another pond that acts as a bio filter, and fishes like tilapia are stocked in it. Nitrogenous wastes are reduced in this bio filter pond and water overflows back to grow-out ponds. Once harvesting is done, the pond bottom shows no signs of smell and black soil accumulation.

## Waste Utilization in Aquaculture

Due to high intake of feed in shrimp and fish ponds, almost 50–60% of the feed is uneaten, which causes high nutrient load because of imbalance of carbon and nitrogen in the system, ultimately leading to water quality deterioration. Since dietary nutrients supplied in feed are not fully recovered and each nutrient contributes to the total feed cost, increasing nutrient utilization efficiency would improve fish or shellfish production economics (32). The increase of nutrient waste in a pond aquaculture system can have adverse implications that limit production and pollute the environment. The BFT can improve water quality in these ponds by regulating carbon and nitrogen through the processes photosynthesis and nitrification (12). It is reported that mullet (*Mugil liza*) were able to lower total suspended solids generated from vannamei production in a BFT system by consuming solids; however, their culture in the same tank resulted in shrimp growth being reduced (116). Furthermore, a biofloc inoculum could be used to modify the nitrifying bacterial community in mullet and shrimp community culture (116). Kim et al. (117), in his study, demonstrated that without any sodium bicarbonate supplementation, the biofloc system demonstrated satisfactory recovery and sustainable water quality management. Heterotrophic bacteria are the most prevalent microbial community members in biofloc that are mostly responsible for generating the structure of biofloc. Similarly, chemoautotrophic nitrifiers are lower in number than other types of bacteria that ultimately results in less nitrogen efflux into a pond ecosystem (117). Actinobacteria also facilitate the development of biofloc, which may be required for secondary protection against fish infections. They may, however, lead to off flavor in fish flesh and pond water (118). In BFT artemia biomass, polychaetes, and semi-moist feed were the most important fresh food sources for broodstock origins in both *Farfantepenaeus brasiliensis* and *Litopenaeus vannamei* (119, 120). In addition, there is no significant difference in spawning performance among females reared in the BFT with or without feed supplementation (121). The use of biofloc can also improve the growth, survival, and reproductive performance of cultured animals (56, 122). However, some species like *Farfantepenaeus duorarum*, in their natural habitat, produce a larger number of eggs per spawning period than those cultured in the biofloc system (123).

Free amino acids, such as alanine, glutamate, arginine, and glycine, which have been identified as possible attractants in shrimp diets (31) and are present in biofloc, are found to be comparable to those reported in commercial shrimp diets (16). This indicates that some aquaculture organisms are likely to recognize biofloc as food particles. According to Avnimelech (39), using the biofloc system in tilapia intensive cultures enhanced nitrogen recovery from 23 to 43%. Second, since the biofloc system employs no or limited water exchange, it is believed that it will use water more efficiently than a traditional system having regular water exchange. Ekasari (17), in his experiment, concluded that regardless of the animal species cultured, nitrogen uptake from biofloc with a particle size of >100 m was determined to be the highest. The ability of the biofloc system to improve nutrient utilization efficiency in aquaculture systems is clearly demonstrated by nutrient conversion by biofloc followed by consumption by cultured animals.

Aquaculture mimicry, also known as aquamimicry, is a technique that involves simulating natural estuary conditions in culture ponds. To improve and sustain water quality, zooplanktons bloom, primarily copepods, and beneficial bacterial populations are established. Copepods can bloom as soon as 2 days after applying FRB, depending on water source, temperature, and previous pond management (113). It refers to the integration of aquatic biology and technology to construct living beings that mimic the nature of aquatic ecosystems for the well-being and development of aquatic animals, with the aim of reviving the shrimp farming sector on a long-term basis. Aquamimicry technology is being used to create a natural estuary environment by encouraging and balancing the growth of natural planktonic communities. Natural feeds found in water resources, such as zooplanktons, artemia, and phytoplanktons, are fed to shrimp using this approach (115). Shrimp do not consume phytoplankton directly. They devour phytoplankton-eating tiny organisms or bacteria that grow on dead phytoplankton cells that gather on the bottom. The culture environment ideally resembles a natural estuary environment with balanced water quality provided by acquired planktons with these biofloc changes (113). As a result, feed consumption and water exchange can be minimized. This technology is proved best for waste utilization, as with the continuous use of carbon sources and probiotics, planktonic growth can be supported.

## Aquamimicry vs. Biofloc Technology

Both aquamimicry and the BFT depends on external carbon source addition. In the biofloc system, in order to maintain floc, the C:N ratio is to be maintained at 15:1 (124). Heterogeneous bacteria then derive carbon from a supplied carbonaceous substrate. However, in the concept of aquamimicry, once stocking of cultured organism is done, the carbon source to be added is dependent on the level of intensification (extensive or intensive) and turbidity level of the water. The BFT is considered as an economical method for aquaculture (12) by acting as live feed for stocked species (21), but a major disadvantage of the technology is the need for continuous and rigorous aeration for suspending generated wastes, which is further metabolized by bacteria for protein generation (105).

The concept of aquamimicry originated from Thailand during a disease outbreak in the early 1990s (115). In aquamimicry, the major vital ingredient used in the system is rice bran, which acts as a carbonaceous source and is easily available in nearby market. Initially, rice bran was used in the culture system but was later replaced by standardized fermented rice having higher efficiency. Rice bran is rich in nutrients but at the same time contains higher fiber contents (125). Therefore, by using the fermentation process lipid, ash, fiber, and phytic acid were successfully removed from rice bran, and the technique was standardized (126). In the Philippines, a technical project was started by JICA to understand and evaluate the benefits of pond-grown natural food. Initially, fish in ponds were fed with rice bran, and compounded feed was given; the calculated feed conversion ratio (FCR) was found to be 1.27, and profitability was 17%. Later in the experiment, rice bran was replaced with fermented rice bran and the profitability rose to around 40%, believing that fermentation increased the protein content (127). In aquamimicry, the production system contains the beneficial zooplanktons and microalgae that are produced in the water, and the system mimics natural estuarine waters. The system does not necessitate the use of any chemical or antibiotics, since rice bran itself provides nutrition to the zooplanktons, and the bacteria present are responsible for the development of pre- and probiotics creating a healthy environment. In this technology, post-harvest pond preparation does not need much effort and attention, since the bottom of the pond reportedly does not have black soil, smell, and sediments and is often ready for next production cycles. In most cases, there are three significant limitations in adopting this aquamimicry technology: first, this idea is extremely difficult to implement in indoor settings; second, this system requires huge treatment ponds; another disadvantage of this method is that the trash produced in the form of excessive sediments is not recyclable and has to be disposed off, which is not the case with BFT.

## Conclusion

The current major crisis the world is facing is ever-increasing seafood demand, water scarcity, and cultivable land resources. In order to overcome these problems, the best solution is intensive sustainable aquaculture. The BFT can serve as an eco-friendly and sustainable effort to not only increase aquaculture production but use minimum land and water resources. For a cultured species to grow and prosper, nutrition plays a vital role, and biofloc acts as best substitute for fishmeal in a diet reducing the FCR and, consequently, feed costs. Biofloc is rich in microbial protein and contains an organic polyhydroxybutyrate polymer that, when introduced with commercial feeds, forms a complete healthy and nutritious food chain and improves growth performance. Advantages of the BFT include improved biosecurity, less feed utilization, lower pathogenic introduction, higher growth and survival, reduced water exchange, and, hence, better productivity. Farmers must be trained in a practical manner about the successful experiences of the BFT as well as the economic benefits. Aquaculture now has a sustainable solution to solve its environmental, social, and economic challenges at the same time that it grows owing to the BFT. Many studies have been carried out to understand the growth and development of herbivorous and detrivorous fishes in biofloc systems. There is a need to conduct more research and experimentation for carnivorous organisms with different carbon sources and ratios to see whether the BFT can provide them with any benefits. Therefore, it is recommended that future studies be conducted in order to provide the most appropriate specialized conditions for many species in the system. Researchers should also be encouraged to improve this technology, and farmers should be scientifically advised to incorporate this technology into their future aquaculture systems. Future research should emphasize on the role and significance of the BFT in persuading farmers to implement it. Organic aquaculture products from BFT ponds should be promoted to customers through suitable supply chains and marketing techniques.

Aquamimicry is a revolutionary concept that simulates natural estuarine conditions by developing copepods that act as supplementary nutrition to a cultured species. It is a more balanced approach that uses both microalgae and biofloc in aquaculture. The concept is best known for reducing the stress associated with fluctuating water quality, and it minimizes favorable environmental conditions to pathogens. Usually, the shrimps produced by this technology are rich in astaxanthin, PUFAs, and amino acids responsible for red coloring in shrimps, which increases the market value by labeling the shrimps as “organic shrimps” (109). The technique can be very fruitful in uplifting the economic status of shrimp farmers by producing disease-free and cost-efficient produce.

## Author Contributions

UN was responsible for conceptualization, methodology, and preparation and writing of the original draft. YM and YS performed visualization, investigation, and supervision. DP helped in critically reviewing the manuscript. All authors contributed to the article and approved the submitted version.

## Funding

The authors acknowledge the financial support from the China Agriculture Research System of MOF and MARA and programs of development of trading company performance appraisal system based on big data platform (6602421707), Rizhao Donggang Hongqi Phase II Modern Fishery Industrial Park (6602420717), and the high-level talent research startup fund of Qingdao Agricultural University (663/1116710).

## Conflict of Interest

The authors declare that the research was conducted in the absence of any commercial or financial relationships that could be construed as a potential conflict of interest. The reviewer SK declared a shared affiliation with several of the authors, UN, DP and YM at the time of review.

## Publisher's Note

All claims expressed in this article are solely those of the authors and do not necessarily represent those of their affiliated organizations, or those of the publisher, the editors and the reviewers. Any product that may be evaluated in this article, or claim that may be made by its manufacturer, is not guaranteed or endorsed by the publisher.
